# Exploring the effects of task complexity and translation anxiety on EFL learners’ translation performance: Evidence from a mixed-design study

**DOI:** 10.1371/journal.pone.0346731

**Published:** 2026-05-06

**Authors:** Xiangyan Zhou, Xiaodong Liu

**Affiliations:** 1 School of Foreign Studies, Hunan University of Humanities, Science and Technology, Loudi, China; 2 College of Foreign Languages, Hunan University, Changsha, China; Stellenbosch University, SOUTH AFRICA

## Abstract

While the impact of task complexity on translation performance has received considerable attention, relatively little research has explored whether affective factors such as translation anxiety moderate this effect. This study investigated the joint influence of task complexity and translation anxiety on English as a foreign language (EFL) learners’ written translation performance using a 2 × 2 mixed design. A total of 106 EFL learners from diverse disciplines in China participated in the study, with 100 included in the final analyses; their translation anxiety was assessed using a Translation Anxiety Scale adapted for written translation. Participants completed two written translation tasks at different complexity levels. Their translation performance was assessed in terms of process efficiency (i.e., total processing time, subjective cognitive effort, and the number of effective revisions) and product quality (i.e., accuracy, fluency, and analytical quality measures). Linear mixed-effects models showed consistent effects of task complexity on translation performance, whereas translation anxiety played a selective and context-dependent role, mainly by moderating the effects of task complexity on some efficiency outcomes. The study develops a conceptual framework that integrates task-related factors and learner factors as predictors of translation performance and offers pedagogical insights for translation teaching.

## Introduction

In some multilingual and bilingual contexts, translation serves as a key tool for language learners, facilitating the development of fundamental language skills and contributing to foreign language acquisition [1]. Reflecting this pedagogical value, translation has gained increasing recognition in formal education systems worldwide. Translation competence has been conceptualized as a multi-componential construct that can be developed through instruction and assessed in educational settings, as reflected in models and frameworks such as the PACTE model and the EMT competence framework [2–3]. In such settings, translation competence is commonly cultivated through two institutional arrangements: foreign language programs that incorporate translation or dedicated translator education programs, where language development and translation learning are often closely intertwined [4]. In China, translation instruction is also provided through public foreign language courses for students learning English as a foreign language (EFL) in non-English-major programs [5].

In translation classrooms, students are encouraged to produce high-quality translations efficiently. However, a trade-off often exists between translation efficiency and quality [6], and different factors can influence different dimensions of learners’ performance in translation tasks [7]. In the current study, a “task” is defined as a goal-directed activity that can be decomposed into subtasks at varying levels of granularity [8]. This definition is well suited for describing and analyzing the structured nature of the translation process, which often involves multiple subtasks. Importantly, the present definition is adopted for translation process research and should be distinguished from the pedagogical definition of “task” in task-based language teaching (TBLT). In TBLT, a task is typically defined as an activity that requires learners to use language and to focus primarily on meaning, and is expected to bear real-world relevance [9–11]. To help learners balance translation efficiency and quality during task execution, it is essential to understand how various factors differentially influence learners’ translation performance across key process and product dimensions.

Psycholinguistic approaches to translation, which focus on the mental processes involved in translation [12], have deepened our understanding of how task-related factors shape translation performance. Specifically, studies suggest that manipulating task design features (e.g., [13–14]) or task implementation conditions (e.g., [15–16]) can shift learners’ attentional focus and the allocation of cognitive resources during task engagement, and ultimately affect their process efficiency and product quality. Within this line of research, task complexity has received particular attention as a salient and operationalizable task feature for explaining variation in translation performance [17]. However, findings remain mixed. A plausible explanation is that task complexity effects are often examined without explicitly modelling learner factors that may shape translation performance. To better account for the mixed effects of task complexity on translation performance, it is important to consider learner factors [9].

Learner factors can be broadly grouped into four categories: sociodemographic (e.g., age, and cultural background), cognitive (e.g., working memory capacity, and reasoning abilities), conative (e.g., motivation, and willingness to communicate), and affective (e.g., anxiety, and enjoyment) [9]. Building on this broader perspective, studies in translation research and broader task performance research have examined how learner factors shape performance outcomes (e.g., [18–23]). Within translation studies, affective variables have received increasing attention [7]. For example, anxiety [16], self-efficacy belief [17], and emotional intelligence [18] have been found to influence various aspects of translation performance, including psychological responses, cognitive resource allocation, and translation quality. Despite this growing body of research, relatively few empirical studies have examined whether affective variables moderate the effects of task complexity on translation performance, particularly when performance is assessed across both process and product dimensions. This study addresses this gap by investigating the joint and differential effects of task complexity and translation anxiety on the process efficiency and product quality of EFL learners’ performance in written translation tasks.

## Literature review

### Investigating the process and product features of translation performance

Translation performance has been investigated through two primary lenses: the product-oriented approach and the process-oriented approach [17]. The product-oriented approach evaluates translation performance based on the quality of the final output, viewing performance as the outcome of translation activities. For instance, Ferdowsi and Razmi [18] assessed interpreting performance by employing oral cloze tests and memory recall tasks, both aimed at assessing output accuracy. In contrast, the process-oriented approach conceptualizes translation as a cognitive activity, evaluating translation performance based on individuals’ behaviors and cognitive processing during task execution. For example, Núñez and Bolaños-Medina [19] examined the influence of students’ intrinsic motivation on translation sub-processes, including problem identification, solution generation, and decision-making.

While both approaches offer valuable insights, an approach that integrates both product and process dimensions is essential for a more comprehensive understanding of translation performance (e.g., [24,25]). However, relatively few studies have adopted this dual-focus approach [26]. Regarding process features, translation efficiency is commonly assessed through indicators such as self-reported cognitive effort, total processing time, and the number of effective revisions (e.g., [17,27,28]). Previous studies have defined cognitive effort as the number of cognitive resources that translators put into task processing [13], and total processing time as the amount of time translators spend completing a translation task [17]. Effective revisions can be conceptualized as revisions that contribute to improving the target text, reflecting translators’ process management during task execution [28,29]. The underlying assumption is that greater efficiency reflects more economical or effective use of cognitive resources, often manifested in faster task completion and, where appropriate, more targeted and effective revision behavior. As for product features, translation accuracy and fluency are often evaluated according to established assessment rubrics (e.g., [17,20]), with accuracy referring to the degree of accurate transfer of source text content, and fluency pertaining to the quality of expression in the target language. Some studies also use text analysis tools to derive analytical metrics for translation quality assessment, such as Coh-Metrix indices that capture textual features like word count and word diversity (e.g., [30]). Accordingly, the present study adopts an integrative approach to examine how different factors jointly influence the process and product dimensions of translation performance.

### Task complexity and translation performance

Task complexity is widely acknowledged as a key factor affecting learner performance in second language acquisition and task-based learning [31]. Two prominent theoretical models have been adopted to explain the effects of task complexity on task performance: Skehan’s Limited Attentional Capacity Model [32], and Robinson’s Cognition Hypothesis [33]. To enable empirical testing and measurement, Robinson further developed the Triadic Componential Framework, which posits that task complexity, task conditions, and task difficulty jointly influence task performance. Within this framework, task complexity refers to task-inherent cognitive demands, whereas task difficulty concerns learners’ perceived task demands, which are shaped by learner factors [31]. The componential framework highlights how interactions between task-related factors and learner factors affect learner performance in language tasks [34]. This line of theoretical development has informed research on translation performance. For instance, based on Robinson’s framework, Wu [14] proposed a three-tier conceptual framework to investigate the impact of task-related factors on learner performance in sight translation, with task complexity operationalized as source text complexity. In his study, Wu [14] suggests that the relationship between task-related factors and translation performance may be mediated by learners’ cognitive, affective, and metacognitive factors. Drawing upon pertinent research, the present study seeks to further extend Robinson’s framework to written translation, aiming to guide future research on how task-related factors (e.g., task complexity) and learner factors (e.g., translation anxiety) interact to influence different dimensions of learners’ written translation performance.

Other studies, although not explicitly grounded in the above-mentioned theoretical frameworks, have also explored how variations in task complexity affect translation processes and/or outcomes (e.g., [13,35–37]). In these studies, task complexity is operationalized using diverse variables, such as source text complexity (e.g., [17,35,38]), text type (e.g., [21,27]), and translation direction (e.g., [36]). However, empirical findings in this area remain inconclusive. When task complexity is operationalized as source text complexity, Zhou et al. [17] reported a significant negative impact on students’ translation efficiency, as indicated by subjective cognitive effort and task duration. In contrast, Hvelplund [35] found no statistically significant effect of text complexity on task duration. Similarly, when task complexity is defined in terms of text type, the findings are also inconclusive. Specialized texts containing more technical terms are often considered more complex [21]. Wang [21] reported longer processing time for the specialized text than for the general text, whereas Jia et al. [27] and Whyatt [36] found no significant difference in processing time between the two text types.

As emphasized in Robinson’s Triadic Componential Framework [31] and Wu’s three-tier conceptual framework [14], learner factors are essential for interpreting task effects on performance, and the mixed findings in translation studies may partly reflect the limited and inconsistent modelling of such factors. Recent task-based research has further highlighted the need to conceptualize learner factors systematically, particularly cognitive and affective variables, as these may shape how task demands are processed by learners [9]. Consistent with this view, prior empirical research in educational contexts suggests that the effects of task complexity may vary as a function of learner factors, including working memory capacity [21], translation experience [38], and anxiety [39]. Building on these theoretical and empirical insights, the present study introduces translation anxiety as a key affective factor that may moderate the effect of task complexity on learners’ performance in written translation tasks.

### Anxiety in translation

Anxiety has emerged as an important affective factor in translation studies, with evidence suggesting that it can significantly predict translation performance (e.g., [22,40,41]). However, there is no consensus on how translation-related anxiety should be defined or measured. Terms such as “stress” and “anxiety” are often used interchangeably (e.g., [16,42]). Moreover, prior research has employed a wide range of instruments to assess participants’ anxiety levels in translation tasks, including trait anxiety scales, state anxiety scales, foreign language classroom anxiety scales, and task-specific anxiety measures (e.g., [15,16,42,43]).

The variation in operationalization has likely contributed to inconsistencies in findings regarding the impact of anxiety on translation performance. For example, Chiang [43] found no relationship between trait anxiety and interpreting scores, but observed a significant negative correlation between foreign language anxiety and interpreting performance. Similarly, Zhu and Ruan [41] reported that interpreting anxiety negatively affected students’ interpreting performance, although their findings did not fully align with Chiang’s. These inconsistencies underscore the need for domain-specific affective measures that are tailored to the task at hand (e.g., [44,45]).

Moreover, most previous studies have adopted a holistic approach to evaluating translation performance (e.g., [41–43]), which may obscure how anxiety affects different dimensions of performance. Yet both theoretical insights and empirical findings suggest that anxiety can exert differential effects on process efficiency and product quality [23]. For instance, Rojo López et al. [15] found that higher levels of trait anxiety impaired translation efficiency but enhanced translation accuracy when no time constraints were imposed. Furthermore, Eysenck et al. [23] argued that the impact of anxiety on academic performance may vary with task complexity. Therefore, when examining the role of anxiety in the relationship between task complexity and translation performance, it is essential to use translation-specific anxiety instruments and to assess both process and product dimensions of performance.

### A conceptual framework for written translation performance

Drawing on the relevant literature, this study proposes a conceptual framework that integrates the predictor variables and assessment dimensions of learner performance in written translation tasks. Specifically, the predictor variables include task-related factors (e.g., task complexity) and learner factors (e.g., translation anxiety), which are posited to influence translation performance. In this study, task complexity is conceptualized as the cognitive demands that translation tasks place on EFL learners and is operationalized as source text complexity, in line with Wu [14]. Translation anxiety is treated as an affective factor that may moderate the relationship between task complexity and translation performance, drawing on Robinson [31] and Wu [14]. Translation performance, as defined by Zhou et al. [17], encompasses two dimensions: process efficiency (e.g., total processing time, subjective cognitive effort, and the number of effective revisions) and product quality (e.g., translation accuracy, fluency, and analytical quality measures). This integrative perspective enables a more comprehensive understanding of learners’ cognitive processes and translation outcomes.

**Fig 1:**
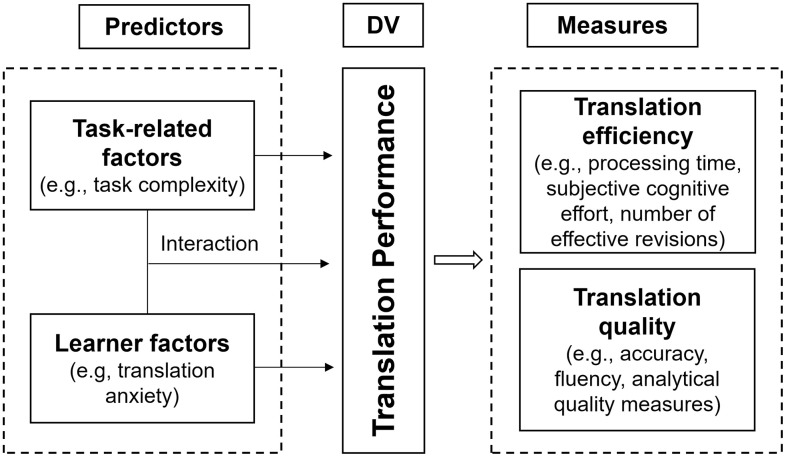
Conceptual framework for written translation performance.

The proposed framework offers an integrative approach to examining how task-related and learner factors interact to shape translation performance, while also highlighting the necessity of assessing both the translation process and product. Furthermore, it addresses methodological gaps in previous research by underscoring the need for context-specific measurement tools, particularly those designed to assess translation anxiety in written translation tasks. Fig 1 illustrates the conceptual framework.

### The present study

Guided by the above-mentioned framework, this study investigates the joint and differential influence of task complexity and translation anxiety on EFL learners’ performance in written translation tasks. Specifically, it examines whether translation anxiety moderates the effects of task complexity on translation efficiency and quality. Translation efficiency is measured by total processing time, subjective cognitive effort, and the number of effective revisions, while translation quality is evaluated in terms of accuracy, fluency, and analytical quality indices generated by Coh-Metrix 3.0. The study seeks to answer the following research questions:

**RQ1.** How does task complexity affect EFL learners’ translation performance, including translation efficiency and quality?

**RQ2.** How does translation anxiety affect EFL learners’ translation performance across tasks of varying complexity levels? Does it moderate the relationship between task complexity and translation performance?

## Methods

The study was reviewed and approved by the Ethics Committee of the School of Foreign Studies at Hunan University of Humanities, Science and Technology (Approval No. 2024–003-SFS). Written consent was obtained from all participants.

### Participants

A total of 106 undergraduate students from various disciplines in China participated in the experiment on a voluntary basis. Participants were recruited via convenience sampling. Among them, 96 were female (91%) and 10 were male (9%), with ages ranging from 19 to 22 years (*M* = 20.51, *SD* = 0.796). All participants were native speakers of Chinese, with approximately 11 years of experience in learning English as a foreign language in formal educational context. Their language proficiency was sufficient to complete the translation tasks independently.

Participants were divided into two groups based on their level of translation anxiety, following the median-split procedure [46]. Specifically, the 50% of students (*N* = 53) with anxiety scores below the median were categorized as the low-anxiety group, while the remaining 50% (*N* = 53) with anxiety scores above the median comprised the high-anxiety group. Their English proficiency was assessed using scores from the College English Test Band 4 (CET-4), a national English proficiency test. Based on published alignment evidence, participants’ scores were approximately mapped to CEFR B1–B2, indicating intermediate to upper-intermediate foreign language proficiency [47]. An independent samples *t*-test confirmed that the two groups differed significantly in translation anxiety (*t* = −11.977, *p* < 0.001, Cohen’s *d* = 2.350), but not in English proficiency (*t* = 1.585, *p* = 0.116, Cohen’s *d* = 0.312).

### Instruments

#### 3.2.1. Translation tasks.

The translation tasks used in the experiment consisted of two informative texts selected and slightly adapted from national English proficiency tests in China. The two source texts addressed the same topic, with lengths ranging between 140 and 160 Chinese characters (punctuation excluded). Readability scores have been widely adopted in translation studies to indicate source text complexity (e.g., [13,48]). In the present study, source text complexity was assessed using four linguistic features generated by the Chinese Readability Index Explorer (CRIE): difficult words (i.e., the total number of words listed in the Academia Sinica database of 3,000 difficult words), sentences with complex semantic categories, personal pronouns, and conjunctions [49]. These features were selected on the basis of prior Chinese readability research, which suggests that texts containing more difficult words, more sentences with complex semantic categories, and more personal pronouns tend to be more complex, whereas texts containing more conjunctions tend to be less complex [50].

Compared with Text II, Text I contained fewer difficult words, fewer sentences with complex semantic categories, and fewer personal pronouns, but more conjunctions. Taken together, these differences indicate that Text I was less complex than Text II (see Table 1 for detailed comparisons). Accordingly, Task 1, based on Text I, was classified as less complex than Task 2, based on Text II. To corroborate this classification, an expert panel was consulted following the procedure described by Sun and Shreve [48]. The panel consisted of two translation instructors, each with over five years of teaching experience, and three professional translators, each with more than ten years of industry experience. These experts independently reviewed the two tasks, evaluated their complexity based on professional judgment, and unanimously agreed that Task 1 was less complex than Task 2.

**Table 1:** Textual features of the two source texts.

Task	Source text	Length (in characters)	Difficult words	Sentences with complex semantic categories	Personal pronouns	Conjunctions
1	Text I	145	26	6	2	6
2	Text II	153	42	7	3	3

#### 3.2.2. Translation anxiety scale.

Following Chiang [43] and García-Pastor and Miller [51], translation anxiety in this study is defined as a situation-specific form of anxiety that reflects an individual’s tendency to experience anxiety in translation contexts. Based on this definition, the state anxiety subscale of the State-Trait Anxiety Inventory (STAI-S) developed by Spielberger et al. [52] was adapted because it specifically measures state anxiety. The STAI-S consists of 20 items rated on a 4-point Likert scale ranging from 1 (not at all) to 4 (very much so). It measures an individual’s anxiety level at the time of completing the questionnaire [52] and has demonstrated good test–retest reliability over a 20-day interval (*r* = 0.86) among college students [53]. To adapt the STAI-S for written translation tasks, minor wording changes were made by incorporating the phrase “written translation” or other contextually appropriate expressions into each item. These changes were intended to preserve the original construct and psychometric properties of the scale while making it suitable for translation-specific use. The resulting instrument, hereafter referred to as the Translation Anxiety Scale, is provided in S1 Table.

A pilot test of the adapted scale was conducted with 131 students from parallel classes at the same university. The adapted scale demonstrated satisfactory internal consistency (Cronbach’s *α* = 0.861), exceeding the commonly accepted threshold of 0.70 [54]. The Kaiser-Meyer-Olkin (KMO) measure (0.832) and Bartlett’s test of sphericity (*χ²*(190) = 1097.944, *p* < 0.001) indicated the data’s suitability for factor analysis. Following the validation procedures recommended by Heggestad et al. [55], content validity was established through expert review by a panel of three experienced translation researchers. The panel confirmed that the contextual modifications were appropriate and did not compromise the scale’s applicability in translation settings. Confirmatory factor analysis (CFA) results showed an acceptable model fit: *χ²*/*df* = 1.75, *CFI* = 0.871, *TLI* = 0.855, *RMSEA* = 0.076 (90% CI [0.061, 0.090]), and *SRMR* = 0.038. Although the *CFI* and *TLI* were slightly below the conventional threshold of 0.90, the majority of model fit indices met the recommended criteria, including *χ²*/*df* < 3, and *RMSEA* and *SRMR* ≤ 0.08 [54,56,57]. The slightly lower CFI and TLI values may be attributed to the relatively small sample size, which has been reported to affect the stability and reliability of fit indices [54,57]. Nonetheless, these results collectively support the reliability and construct validity of the adapted scale for measuring translation anxiety in written translation tasks.

#### 3.2.3. NASA task load index questionnaire.

**Fig 2:**
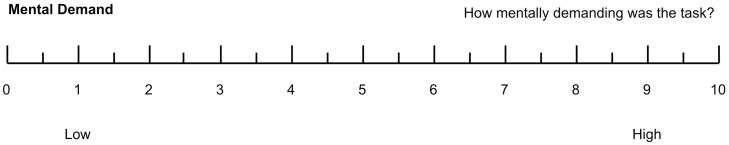
Mental demand subscale in the adapted NASA-TLX questionnaire.

In this study, participants’ subjective cognitive effort was assessed using a questionnaire adapted from the NASA Task Load Index (NASA-TLX), originally developed by Hart and Staveland [58]. This adapted version includes four subscales: mental demand, effort, performance, and frustration, and has demonstrated good reliability in measuring the cognitive resources allocated during translation tasks (e.g., [48,59,60]). For all subscales except performance, higher scores indicate greater cognitive effort. Following the approach of Yuan [60], scores on the performance subscale were reverse coded, as better performance typically corresponds to lower cognitive effort. The overall cognitive effort score was calculated as the mean of the four subscale scores. Fig 2 illustrates the mental demand subscale as an example. The adapted NASA-TLX questionnaire is provided in S2 Table.

#### 3.2.4. Translation quality assessment guidelines.

Two translation teachers from China, each with over five years of experience in translation teaching and assessment, were invited to evaluate the participants’ translations using assessment criteria adapted from Waddington’s (2001) rubric [61]. The rubric employed a five-level rating scale, with each level including two possible scores. This allows raters to make finer distinctions between higher- and lower-quality performances within the same category. The rubric used for human rating of translation quality is provided in S3 Table. Both raters received pre-assessment training to ensure consistent application of the rubric. The training involved familiarizing themselves with the assessment criteria, followed by practice sessions in which they independently scored six translations produced by three students. Afterwards, they engaged in a consensus-building process to compare their scores and negotiate a final agreed mark for each translation. The negotiation approach has been shown to reduce rater bias [62] and is considered effective in translation quality assessment [63]. Once the raters had reached a consensus on the assessment criteria, they proceeded to assess the remaining translations using the same procedure. Any subsequent scoring discrepancies were resolved through consultation with a third expert who had over ten years of professional translation experience.

In addition to human ratings, Coh-Metrix 3.0 was used to provide an analytical assessment of translation quality. This tool has been used in previous studies to analyze textual features relevant to translation quality (e.g., [30]). To obtain a more comprehensive picture of translation quality, three textual features were selected for analysis based on prior research (e.g., [30,64]): word length, word diversity, and the incidence of causal connectives (per 1,000 words). These features have been associated with important aspects of text quality, as they reflect lexical sophistication, lexical diversity, and cohesion. By incorporating these analytical quality measures, the study complements the holistic human ratings and enables a more fine-grained evaluation of translation quality. A detailed description of the textual features used for analytical quality assessment is provided in S4 Table.

### Procedure

**Fig 3:**
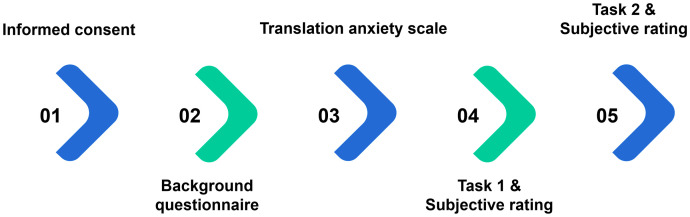
Flowchart of the experimental procedure.

Participants first completed a background questionnaire and the Translation Anxiety Scale before proceeding with the main experimental tasks. During the experiment, they completed two translation tasks on a computer, while screen capture software (pre-installed on each computer) recorded their task duration and translation behaviors. To assess subjective cognitive effort, they completed the adapted NASA-TLX questionnaire after each translation task. The order of the two translation tasks was pseudo-randomized, ensuring that participants alternated between them, thus minimizing sequencing effects. To preserve ecological validity and simulate authentic translation scenarios, the tasks were not time-limited, and participants were permitted to consult online resources during task completion. Fig 3 presents a flowchart of the experimental procedure.

### Data collection and analysis

To investigate how task complexity and translation anxiety affect participants’ translation performance, a quantitative design was adopted. Data on translation efficiency and quality were collected through screen recordings, self-report measures, and translation quality assessments. All analyses were conducted in IBM SPSS Statistics 26, including descriptive statistics and linear mixed-effects models (LMMs). Linear mixed-effects modelling, an extension of multiple regression, is particularly suitable for naturalistic experimental designs because it allows for statistical control of numerous variables that cannot be fully controlled in such environments [59].

Each screen recording was imported into ELAN 6.9 and annotated based on translation phases. According to Dragsted and Carl [29], the translation process consists of three phases: planning, where the translator familiarizes themselves with the source text and plans the translation; drafting, the stage of producing the target text; and monitoring, which involves revising and improving the translation output. Their framework provides a basis for segmenting and analyzing the temporal progression of each task, enabling the derivation of phase duration measures and the number of effective revisions made during the monitoring phase. Following O’Connor and Joffe [65], annotation was independently conducted by two trained coders on 15% of the dataset to assess inter-coder reliability. This subset coding yielded a high Cohen’s kappa of 0.95, indicating excellent agreement. Any discrepancies were resolved through discussion and consensus, after which the remaining 85% of the data were coded by a primary coder. This approach follows established observational coding procedures designed to ensure reliability while maintaining analytic efficiency [65].

Regarding the two dimensions of translation performance, we constructed eleven LMMs with task complexity and translation anxiety as fixed effects and participants as a random effect. In IBM SPSS Statistics 26, participant ID was specified as the Subject variable and included in the LMMs as a random intercept to account for repeated observations nested within participants. The dependent variables of these LMMs consisted of various measures representing translation efficiency and quality, with each model including one dependent variable. During data analysis, we first tested the main effects of task complexity and translation anxiety, as well as the interaction between them. In reporting the results, main effects and interactions were first reported in terms of statistical significance, followed by parameter estimates where appropriate. Significant interactions were further examined using simple effects analyses [66]. The significance level was set at *p* = 0.05, and Cohen’s *f*^*2*^ was employed to assess effect sizes. Following Cohen [67], Cohen’s *f*^*2*^ values were interpreted as small (0.02 ≤ *f*^*2*^ < 0.15), medium (0.15 ≤ *f*^*2*^ < 0.35), and large (*f*^*2*^ ≥ 0.35).

## Results

Preliminary data screening identified 100 complete cases and 6 cases with missing data. Examination of the screen recordings showed that 6 students had not fully recorded their translation process and were therefore excluded from the final analyses. The final sample thus comprised 100 students, including 49 in the low-anxiety group and 51 in the high-anxiety group.

### Influence on translation efficiency measures

The first set of LMMs focused on translation efficiency, with total processing time and phase durations (in seconds) as dependent variables. Overall, task complexity showed significant main effects on total processing time (*p* < 0.001, *f*^*2*^ = 0.300), drafting time (*p* < 0.001, *f*^*2*^ = 0.373), and monitoring time (*p* < 0.05, *f*^*2*^ = 0.011), but not on planning time (*p* = 0.167). Translation anxiety showed no significant main effects on total processing time, planning time, drafting time, or monitoring time (*p* = 0.905, 0.508, 0.470, and 0.130, respectively). The interaction between task complexity and translation anxiety was significant only for total processing time (*p* < 0.05, *f*^*2*^ = 0.007), but not for planning time, drafting time, or monitoring time (*p* = 0.199, 0.125, and 0.604, respectively). Follow-up parameter estimates indicated that drafting time was significantly longer in the complex task than in the simple task (*b* = −281.314, *SE* = 27.044, *t* = −10.402, *p* < 0.001), whereas monitoring time was significantly longer in the simple task than in the complex task (*b* = 39.490, *SE* = 18.821, *t* = 2.098, *p* < 0.05). For total processing time, the parameter estimate for the interaction term indicated that the effect of task complexity varied by anxiety group (*b* = −85.031, *SE* = 41.486, *t* = −2.050, *p* < 0.05).

**Fig 4:**
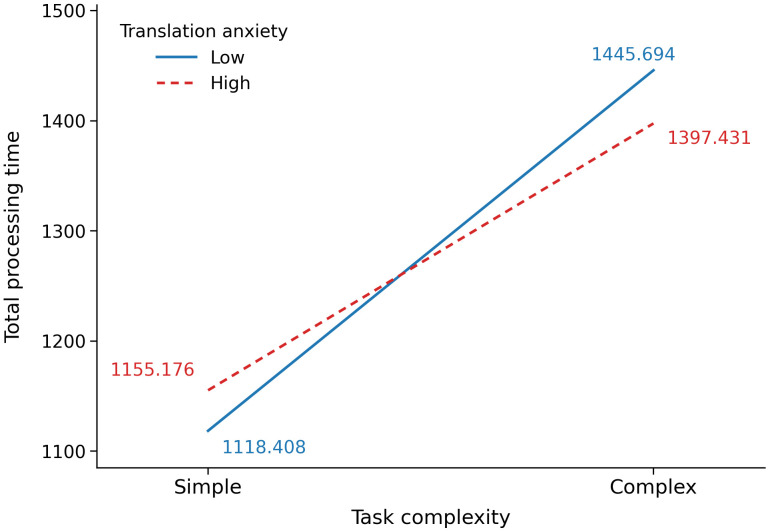
Interaction effect of task complexity and translation anxiety on total processing time.

Simple effects analyses for total processing time further showed that the complex task resulted in significantly longer total processing time than the simple task for both the low-anxiety group (complex: *M* = 1445.694, *SD* = 301.055; simple: *M* = 1118.408, *SD* = 253.151; *p* < 0.001) and the high-anxiety group (complex: *M* = 1397.431, *SD* = 245.830; simple: *M* = 1155.176, *SD* = 243.373; *p* < 0.001). Notably, the task complexity effect was larger in the low-anxiety group (327.286 seconds) than in the high-anxiety group (242.255 seconds), consistent with the significant interaction between task complexity and translation anxiety in the corresponding LMM. However, no significant between-group differences were found within either task condition (complex: *p* = 0.358; simple: *p* = 0.483). Overall, these findings indicate that translation anxiety moderated the effect of task complexity on total processing time (see Fig 4 for details).

**Fig 5:**
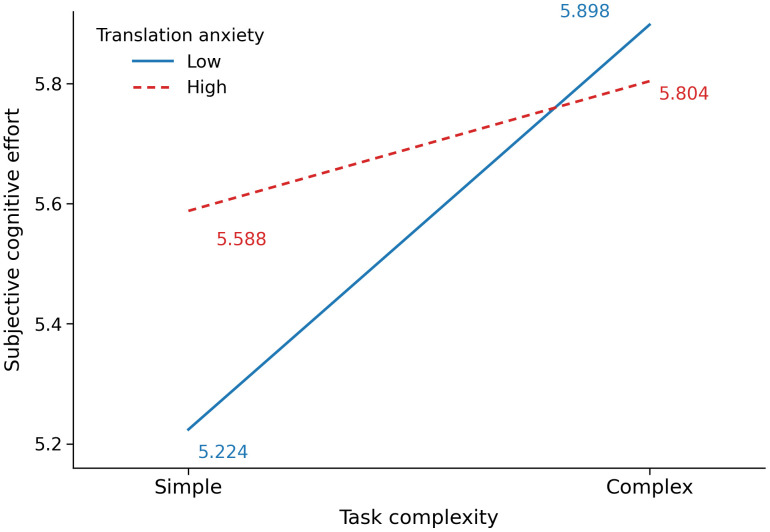
Interaction effect of task complexity and translation anxiety on subjective cognitive effort.

Translation efficiency was also measured by subjective cognitive effort. Overall, task complexity showed a significant main effect (*p* < 0.001, *f*^*2*^ = 0.064), whereas translation anxiety did not (*p* = 0.350). In addition, the interaction between task complexity and translation anxiety was significant (*p* < 0.05, *f*^*2*^ = 0.018). The parameter estimate for the interaction term indicated that the effect of task complexity differed significantly by anxiety group (*b* = −0.458, *SE* = 0.200, *t* = −2.290, *p* < 0.05). Simple effects analyses further revealed that task complexity significantly affected subjective cognitive effort, but only among the low-anxiety participants. Specifically, in the low-anxiety group, participants reported significantly greater cognitive effort in the complex task (*M* = 5.898, *SD* = 0.860) than in the simple task (*M* = 5.224, *SD* = 0.860) at *p* < 0.001. In contrast, no significant difference was observed in the high-anxiety group (complex: *M* = 5.804, *SD* = 0.807; simple: *M* = 5.588, *SD* = 0.963; *p* = 0.127). Furthermore, translation anxiety significantly influenced subjective cognitive effort, but only in the simple task. During the simple task, the low-anxiety group reported significantly less effort than the high-anxiety group (*p* < 0.05), whereas no significant difference was found in the complex task (*p* = 0.592). These results indicate that translation anxiety moderated the effect of task complexity on subjective cognitive effort (see Fig 5 for details).

In addition, translation efficiency was measured by the number of effective revisions made during the monitoring stage. Overall, task complexity, translation anxiety, and their interaction did not significantly affect the number of effective revisions during monitoring (*p* = 0.183, 0.071, and 0.721, respectively). Descriptive analyses suggested that the number of effective revisions was broadly comparable across task conditions, with the low-anxiety group making slightly fewer revisions in the complex task (*M* = 1.837, *SD* = 3.016) than in the simple task (*M* = 2.041, *SD* = 2.525) and the high-anxiety group showing a similar pattern (complex: *M* = 1.020, *SD* = 1.463; simple: *M* = 1.373, *SD* = 1.865). This result was consistent with the non-significant LMM results.

### Influence on translation quality measures

Translation quality was assessed in terms of accuracy, fluency, and analytical quality measures. For accuracy, task complexity showed a significant main effect (*p* < 0.001, *f*^*2*^ = 0.658), whereas translation anxiety did not (*p* = 0.613). The interaction between task complexity and translation anxiety was also not significant (*p* = 0.804). Follow-up parameter estimates indicated that translation accuracy was significantly lower in the complex task than in the simple task (*b* = 1.667, *SE* = 0.134, *t* = 12.466, *p* < 0.001). Across anxiety groups, the observed means were 5.290 (*SD* = 0.743) for the complex task and 6.980 (*SD* = 1.287) for the simple task. Table 2 presents the descriptive statistics for accuracy.

**Table 2:** Means and standard deviations of accuracy.

Factor	Task complexity	Translation anxiety	*N*	Mean	*SD*
Task complexity	Simple	/	100	6.980	1.287
	Complex	/	100	5.290	0.743
Translation anxiety	/	Low	49	6.184	0.882
	/	High	51	6.088	0.994
Task complexity * Translation anxiety	Simple	Low	49	7.041	1.224
		High	51	6.922	1.354
	Complex	Low	49	5.327	0.658
		High	51	5.255	0.821

Translation quality was also assessed in terms of fluency. For fluency, task complexity showed a significant main effect (*p* < 0.001, *f*^*2*^ = 0.468), whereas translation anxiety did not (*p* = 0.358). The interaction between task complexity and translation anxiety was also not significant (*p* = 0.471). Follow-up parameter estimates indicated that translation fluency was significantly lower in the complex task than in the simple task (*b* = 1.745, *SE* = 0.109, *t* = 16.052, *p* < 0.001). Across anxiety groups, the observed means were 4.390 (*SD* = 1.109) for the complex task and 6.080 (*SD* = 1.376) for the simple task. Table 3 presents the descriptive statistics for fluency.

**Table 3:** Means and standard deviations of fluency.

Factor	Task complexity	Translation anxiety	*N*	Mean	*SD*
Task complexity	Simple	/	100	6.080	1.376
	Complex	/	100	4.390	1.109
Translation anxiety	/	Low	49	5.347	1.091
	/	High	51	5.127	1.276
Task complexity * Translation anxiety	Simple	Low	49	6.163	1.280
		High	51	6.000	1.470
	Complex	Low	49	4.531	0.981
		High	51	4.255	1.214

LMMs were also conducted to examine the effects of task complexity and translation anxiety on analytical quality measures. Overall, task complexity showed significant main effects on word length (*p* < 0.001, *f*^*2*^ = 1.660), word diversity (*p* < 0.001, *f*^*2*^ = 0.482), and the incidence of causal connectives (*p* < 0.001, *f*^*2*^ = 1.000). Translation anxiety showed no significant main effects on word length, word diversity, or the incidence of causal connectives (*p* = 0.273, 0.601, and 0.065, respectively). No significant interactions between task complexity and translation anxiety were observed for the three measures (*p* = 0.070, 0.371, and 0.742, respectively). Follow-up parameter estimates for task complexity indicated that, compared with the complex task, the simple task featured significantly longer word length (*b* = 0.474, *SE* = 0.032, *t* = 14.712, *p* < 0.001), higher word diversity (*b* = 0.068, *SE* = 0.008, *t* = 8.153, *p* < 0.001), and a higher incidence of causal connectives (*b* = 21.278, *SE* = 2.183, *t* = 9.745, *p* < 0.001). Across anxiety groups, the mean values for the simple and complex tasks were 4.955 (*SD* = 0.213) and 4.439 (*SD* = 0.192) for word length, 0.822 (*SD* = 0.051) and 0.749 (*SD* = 0.054) for word diversity, and 34.273 (*SD* = 12.306) and 12.492 (*SD* = 9.675) for the incidence of causal connectives, respectively. Table 4 presents the descriptive statistics for analytical quality measures.

**Table 4:** Means and standard deviations of analytical quality measures.

Factor	Task complexity	Translation anxiety	*N*	Word length		Word diversity		Incidence of causal connectives	
**Mean**	***SD***	**Mean**	***SD***	**Mean**	***SD***
Task complexity	Simple	/	100	4.955	0.213	0.822	0.051	34.273	12.306
	Complex	/	100	4.439	0.192	0.749	0.054	12.492	9.675
Translation anxiety	/	Low	49	4.716	0.182	0.788	0.043	21.904	7.996
	/	High	51	4.679	0.148	0.783	0.045	24.803	7.432
Task complexity * Translation anxiety	Simple	Low	49	4.995	0.237	0.827	0.048	33.057	13.699
		High	51	4.917	0.180	0.817	0.054	35.442	10.809
	Complex	Low	49	4.436	0.205	0.748	0.056	10.751	8.607
		High	51	4.442	0.181	0.749	0.054	14.164	10.412

### Correlation analysis of translation quality and efficiency measures

As shown in Table 5, Pearson correlation analysis revealed significant negative relationships between all quality measures and two of the three efficiency measures (i.e., total processing time and subjective cognitive effort). Specifically, translation accuracy was negatively correlated with total processing time (*p* < 0.001) and subjective cognitive effort (*p* = 0.001). A similar pattern was observed for translation fluency. Analytical quality measures (i.e., word length, word diversity, and the incidence of causal connectives) also showed significant negative correlations with processing time and cognitive effort. In contrast, none of the quality measures showed a significant correlation with the number of effective revisions. Based on Cohen’s guidelines [67], these correlation coefficients represent small to medium effect sizes (small: 0.1 < |*r*| < 0.3; medium: 0.3 < |*r*| < 0.5), indicating small-to-moderate negative associations overall.

**Table 5:** Correlation coefficients between translation quality and efficiency measures.

	total processing time	Subjective cognitive effort	Number of effective revisions
Accuracy	*r* = −0.319, *p* < 0.001	*r* = −0.225, *p* = 0.001	*r* = 0.136, *p* = 0.055
Fluency	*r* = −0.406, *p* < 0.001	*r* = −0.245, *p* < 0.001	*r* = 0.044, *p* = 0.532
Word length	*r* = −0.293, *p* < 0.001	*r* = −0.236, *p* = 0.001	*r* = 0.133, *p* = 0.060
Word diversity	*r* = −0.233, *p* = 0.001	*r* = −0.318, *p* < 0.001	*r* = 0.043, *p* = 0.548
Incidence of causal connectives	*r* = −0.255, *p* < 0.001	*r* = −0.194, *p* < 0.01	*r* = 0.120, *p* = 0.091

## Discussion

This study aimed to investigate the effects of task complexity and translation anxiety on EFL learners’ integrated translation performance, as well as to explore whether translation anxiety moderates the effect of task complexity. Our analysis not only confirmed some previous findings but also provided new insights through data triangulation.

### Task complexity effects

The first research question examined the influence of task complexity on translation efficiency and quality. Overall, the findings suggest that task complexity had a consistent influence on translation efficiency and quality. This pattern is broadly consistent with the findings of Zhou et al. [68], who reported that ambiguous words, treated as an indicator of higher task complexity, were processed more slowly and less accurately than unambiguous words.

Consistent with this overall pattern, the complex task was associated with longer processing time compared to the simple task. A phase-based analysis showed that this difference was particularly evident during the drafting phase, suggesting that increased task demands primarily burdened the stage of target text production. Similar findings were reported by Zhou et al. [17]. One plausible explanation is that increased drafting time may reflect differences in participants’ process management, as task complexity shapes how learners process information and allocate cognitive resources during translation (e.g., [13–14]). For example, under more demanding task conditions, learners may allocate additional time to verifying their choices, which can extend drafting time. As Cui and Zheng [69] observed, more cognitively demanding tasks tend to prompt learners to spend more time consulting reference materials to meet increased information needs, particularly during drafting.

For subjective cognitive effort, task complexity showed a significant main effect, but this effect varied by learners’ anxiety level. Specifically, greater effort was invested in the complex task than in the simple task only among the low-anxiety participants. This finding is partially consistent with Liu and Zheng [59], who reported that students exerted greater effort on more challenging tasks to maintain translation quality. One possible explanation is that learners may increase cognitive effort as task demands rise [70]. However, because this pattern was not observed across the full sample, the effect of task complexity on subjective cognitive effort may depend on learners’ anxiety level. This group-specific pattern is discussed further in the following section.

Lastly, the complex task led to lower translation quality. A plausible explanation lies in students’ limited cognitive capacity [70]. When task demands exceed a manageable threshold, cognitive overload can occur, making it difficult for learners to coordinate multiple subtasks and maintain task performance [59]. Consistent with this, Wu [14] noted that complex syntactic structures place greater demands on cognitive resources during the translation process, which can in turn reduce the quality of the final output. These findings highlight the importance of paying closer attention to the translation process, as difficulties encountered during the process are likely to affect the quality of the product. Understanding where students encounter challenges in complex tasks can help teachers provide targeted support to improve process efficiency, product quality, or both. Additionally, correlation analysis revealed a negative relationship between translation efficiency and quality across most indicators. This finding is consistent with Mossop et al.’s [6] claim of a trade-off between translation efficiency and quality, suggesting that when cognitive resources are limited, gains in one may come at the expense of the other. Notably, the efficiency–quality trade-off was more apparent for total processing time and subjective cognitive effort than for the number of effective revisions, indicating that in the present data, time- and effort-based indicators were more sensitive to task demands than revision behavior.

### Translation anxiety effects

The second research question examined the effects of translation anxiety on translation performance and its potential moderating role in the relationship between task complexity and translation performance. Overall, translation anxiety showed selective, context-dependent effects on translation efficiency. Although no significant main effects of translation anxiety were found, it significantly moderated the effects of task complexity on two of the three efficiency indicators (e.g., total processing time and subjective cognitive effort). This pattern underscores the importance of assessing process efficiency using multiple indicators, as task-related factors and learner factors may affect different efficiency measures in different ways (e.g., [17]).

Specifically, although the between-group differences in total processing time were not significant within either task condition, the descriptive analyses showed that the low-anxiety group spent more time processing the complex task than the high-anxiety group. One plausible interpretation is that the two groups differed in the flexibility of their strategy use during problem solving under increased task demands. When encountering translation problems, learners may draw on internal resources (e.g., inference and prior knowledge) or external resources (e.g., online dictionaries and reference materials), or alternate between the two [2]. In the present study, the longer total processing time observed for the low-anxiety group in the complex task may reflect more flexible strategy use during problem solving, with more frequent shifts between internal and external support potentially requiring additional time. This account is partially consistent with Cui and Zheng [69], who found that increased task complexity was associated with more time spent consulting online resources among students. By contrast, the high-anxiety participants may have shown less flexible strategy use or terminated verification earlier, possibly because some attentional resources were diverted to anxiety-related concerns, a pattern compatible with attentional control theory [23]. This interpretation remains tentative and warrants further examination using more direct process-tracing data on strategy use and resource consultation (e.g., eye-tracking or keystroke logging).

A different pattern emerged for subjective cognitive effort. The high-anxiety group reported significantly higher subjective cognitive effort than the low-anxiety group in the simple task. This between-group difference is consistent with earlier findings suggesting that high-anxiety individuals often expend more mental effort to achieve comparable levels of performance because anxiety-related thoughts consume cognitive resources [23]. At the same time, the complexity-related increase in cognitive effort was statistically evident only among the low-anxiety participants, whereas the high-anxiety group showed no significant change across the two tasks. One possible explanation is that anxiety may have limited high-anxiety learners’ capacity to cope with increasing task demands by investing additional effort [23]. Another possible explanation is that the two groups may also have differed in their willingness to invest additional effort under more demanding conditions, as learner attitude has been described as a strong “determinant of the actual effort applied” [70].

The results also revealed that translation anxiety moderated the effects of task complexity on total processing time and subjective cognitive effort. Overall, these findings suggest that anxiety may shape how learners experience task demands (as reflected in subjective cognitive effort) and allocate time during task performance (as reflected in total processing time), although this moderating role appears to be measure-specific and was not observed for the revision-based efficiency indicator. Taken together, these findings provide partial support for Tobias’ [39] conclusion and further highlight the importance of considering learner factors, such as translation anxiety, when investigating the impact of task complexity on translation performance.

Finally, across accuracy, fluency, and analytical quality measures, neither the main effect of translation anxiety nor its interaction with task complexity reached statistical significance, which differs from some previous studies (e.g., [15,41]). However, such comparisons should be made with caution due to differences between those studies and the present one. First, Rojo López et al. [15] used the State-Trait Anxiety Inventory developed by Spielberger et al. [52] to measure participants’ anxiety levels, whereas translation anxiety in this study was measured using the Translation Anxiety Scale, a tool specifically adapted for written translation tasks. Previous research has suggested that differences in measurement tools may partly explain the mixed findings regarding the relationship between anxiety and task performance (e.g., [40,45]). Despite calls for the development and use of anxiety instruments tailored to specific tasks [43,45], many recent studies on translation anxiety have relied on general anxiety scales (e.g., [15–16]). Second, Zhu and Ruan [41] assessed students’ product quality using a holistic score that nonetheless took accuracy, fluency, and time management into account. In contrast, the current study examined participants’ total processing time, subjective cognitive effort, the number of effective revisions, accuracy, fluency, and analytical quality measures independently. Future studies might yield more comparable results if they adopt a widely recognized, context-specific instrument for measuring translation anxiety, and assess translation performance across multiple dimensions.

## Conclusion

This study investigated the role of task complexity and translation anxiety in EFL learners’ written translation performance by using a mixed design and an integrative assessment that captured both process efficiency and product quality. Overall, task complexity exerted consistent effects on both efficiency and quality outcomes. Translation anxiety showed a selective moderating role that was most evident in total processing time and subjective cognitive effort, whereas such moderation was not observed for translation quality.

The findings of this study have both pedagogical implications and theoretical contributions. Pedagogically, the study provides valuable guidance for translation instructors seeking to enhance students’ translation performance through strategic adjustment of task complexity and effective anxiety management. Given that higher task complexity significantly increases total processing time and subjective cognitive effort, it is recommended to introduce complex tasks with appropriate scaffolding. Examples of such scaffolding include the guided use of bilingual dictionaries, parallel corpora, and machine translation tools. Moreover, as anxiety represents a significant affective factor in the EFL context, supporting students in regulating their anxiety may help improve their task performance. Explicit training in emotion regulation strategies may help students reduce cognitive interference caused by anxiety-related thoughts, as suggested by Zhu and Ruan [41]. In particular, because anxiety-related differences were clearly observed in subjective cognitive effort, instructors may consider incorporating low-stakes practice and reflective activities to help students calibrate effort investment across tasks of varying complexity. These measures align with previous studies advocating the development of comprehensive student support systems (e.g., [71]).

Theoretically, this study makes three key contributions. First, it proposes a conceptual framework that integrates task-related factors and learner factors to explain their joint and differential influence on translation performance, addressing a notable gap in previous research that has rarely examined such interactions [14]. Second, the framework defines translation performance as a multidimensional construct encompassing both process efficiency and product quality, thereby responding to the call for research integrating translation process and product [26]. Third, the study adapted and provided initial validation evidence for a translation anxiety scale tailored for written translation tasks, offering a domain-specific tool for future research on anxiety in translation contexts.

Despite these contributions, several limitations warrant consideration. First, in terms of external validity, the study focused on a single genre (informative texts) and one language pair (Chinese–English), and the sample was drawn from a relatively homogeneous cohort (predominantly female EFL students in China). Future research could strengthen generalizability of the findings by examining a wider range of text genres and language pairs and recruiting more demographically diverse and gender-balanced participants across instructional contexts. A second limitation concerns measurement and process evidence. Although the Translation Anxiety Scale was tailored for written translation tasks and underwent initial validation (internal consistency analysis, expert review, and an initial confirmatory factor analysis), further large-scale validation with larger and more diverse samples is warranted to confirm its robustness, factorial structure, and construct validity in translation-specific contexts. Regarding process measurement, the present study relied primarily on processing time, self-reported cognitive effort, and the number of effective revisions as indicators of process efficiency. Future work could strengthen process evidence by complementing these indicators with more objective and fine-grained data derived from process-tracing tools (e.g., keystroke logging and eye-tracking). Finally, future studies may enhance explanatory power by modelling translation anxiety as a continuous construct, which may improve sensitivity to subtle effects and interactions, and by testing potential mediating variables (e.g., translation strategy use and emotion regulation) to clarify the mechanisms linking task-related factors, learner factors, and translation performance.

Overall, this study advances an integrative assessment of EFL learners’ written translation performance by linking task complexity and translation anxiety within a conceptual framework. By examining translation performance across both process efficiency and product quality, the findings offer a basis for more evidence-informed task design and learner support in translation training and provide a foundation for future empirical work on how task-related factors and learner factors jointly and differentially shape translation performance.

## Supporting information

S1 Table Translation anxiety scale. (DOCX)

S2 Table Adapted NASA Task Load Index questionnaire. (DOCX)

S3 Table Rubric for human rating of translation quality. (DOCX)

S4 Table Textual features selected for analytical quality assessment. (DOCX)

S5 File Metadata. (XLSX)
