# Supplementary material for: Exploring the effects of task complexity and translation anxiety on EFL learners’ translation performance: Evidence from a mixed-design study
Source: PLoS One. 2026 May 6;21(5):e0346731. doi: 10.1371/journal.pone.0346731 (PMC13148665; doi:10.1371/journal.pone.0346731)
Supplement: S1 Table — (DOCX) [file pone.0346731.s001.docx]

**S1 Table. Translation anxiety scale**

| **Item** | **Statement** |
| --- | --- |
| 1 | I feel calm about written translation. (Reverse scored) |
| 2 | I feel secure about written translation. (Reverse scored) |
| 3 | I am tense in written translation. |
| 4 | I feel strained about written translation. |
| 5 | I feel at ease about written translation. (Reverse scored) |
| 6 | I feel upset about written translation. |
| 7 | I am worried about possible misfortunes in written translation. |
| 8 | I feel satisfied with written translation. (Reverse scored) |
| 9 | I feel frightened about written translation. |
| 10 | I feel comfortable with written translation. (Reverse scored) |
| 11 | I feel self-confident about written translation. (Reverse scored) |
| 12 | I feel nervous in written translation. |
| 13 | I am jittery in written translation. |
| 14 | I feel indecisive during written translation. |
| 15 | I am relaxed in written translation. (Reverse scored) |
| 16 | I feel content with written translation. (Reverse scored) |
| 17 | I am worried about written translation. |
| 18 | I feel confused about written translation. |
| 19 | I feel steady during written translation. (Reverse scored) |
| 20 | I feel pleasant during written translation. (Reverse scored) |

**Note.** Reverse-scored items are indicated in parentheses.
