# Supplementary material for: Exploring the effects of task complexity and translation anxiety on EFL learners’ translation performance: Evidence from a mixed-design study
Source: PLoS One. 2026 May 6;21(5):e0346731. doi: 10.1371/journal.pone.0346731 (PMC13148665; doi:10.1371/journal.pone.0346731)
Supplement: S2 Table — (DOCX) [file pone.0346731.s002.docx]

**S2 Table. Adapted NASA Task Load Index questionnaire**

| **Dimension** | **Question** | **Scale** |
| --- | --- | --- |
| Mental Demand | How mentally demanding was the task? | 0 (Low) to 10 (High) |
| Effort | How hard did you have to work to accomplish your level of performance? | 0 (Low) to 10 (High) |
| Frustration | How insecure, discouraged, irritated, stressed, and annoyed were you? | 0 (Low) to 10 (High) |
| Performance | How successful were you in accomplishing what you were asked to do? | 0 (Good) to 10 (Poor) |

**Note.** This adapted version included four dimensions: mental demand, effort, frustration, and performance.
