# Supplementary material for: Exploring the effects of task complexity and translation anxiety on EFL learners’ translation performance: Evidence from a mixed-design study
Source: PLoS One. 2026 May 6;21(5):e0346731. doi: 10.1371/journal.pone.0346731 (PMC13148665; doi:10.1371/journal.pone.0346731)
Supplement: S3 Table — (DOCX) [file pone.0346731.s003.docx]

**S3 Table. Rubric for human rating of translation quality**

| **Measure** | **Rating scale** | **Mark** |
| --- | --- | --- |
| **Accuracy** | Level 5:  Complete transfer of ST information; only minor revision needed to reach professional standard. | 9, 10 |
| **Accuracy** | Level 4:  Almost complete transfer; there may be one or two insignificant inaccuracies; requires a certain amount of revision to reach professional standard. | 7, 8 |
| **Accuracy** | Level 3:  Transfer of the general idea(s) but with a number of lapses in accuracy; needs considerable revision to reach professional standard. | 5, 6 |
| **Accuracy** | Level 2:  Transfer undermined by serious inaccuracies; thorough revision required to reach professional standard. | 3, 4 |
| **Accuracy** | Level 1:  Totally inadequate transfer of ST content; the translation is not worth revising. | 1, 2 |
| **Fluency** | Level 5:  Almost all the translation reads like a piece originally written in English. There may be minor lexical, grammatical or spelling errors. | 9, 10 |
| **Fluency** | Level 4:  Large sections read like a piece originally written in English. There are a number of lexical, grammatical or spelling errors. | 7, 8 |
| **Fluency** | Level 3:  Certain parts read like a piece originally written in English, but others read like a translation. There are a considerable number of lexical, grammatical or spelling errors. | 5, 6 |
| **Fluency** | Level 2:  Almost the entire text reads like a translation; there are continual lexical, grammatical or spelling errors. | 3, 4 |
| **Fluency** | Level 1:  The candidate reveals a total lack of ability to express himself/herself adequately in English. | 1, 2 |
