# Supplementary material for: Exploring the effects of task complexity and translation anxiety on EFL learners’ translation performance: Evidence from a mixed-design study
Source: PLoS One. 2026 May 6;21(5):e0346731. doi: 10.1371/journal.pone.0346731 (PMC13148665; doi:10.1371/journal.pone.0346731)
Supplement: S4 Table — (DOCX) [file pone.0346731.s004.docx]

**S4 Table. Textual features selected for analytical quality assessment**

| **Feature category** | **Indicator** | **Measure** | **Description** |
| --- | --- | --- | --- |
| Lexical | DESWLlt | Word length | The average number of letters for all the words in a text (with longer words lower in frequency or familiarity). |
| Lexical | LDTTRc | Word diversity | The variety of unique words that occur in a text. |
| Textual | CNCCaus | Causal connectives | The incidence of causal connectives (per 1,000 words) in a text. |
